# Multimodal fusion of multiple rest fMRI networks and MRI gray matter via parallel multilink joint ICA reveals highly significant function/structure coupling in Alzheimer's disease

**DOI:** 10.1002/hbm.26456

**Published:** 2023-08-22

**Authors:** K. M. Ibrahim Khalilullah, Oktay Agcaoglu, Jing Sui, Tülay Adali, Marlena Duda, Vince D. Calhoun

**Affiliations:** ^1^ Tri‐institutional Center for Translational Research in Neuroimaging and Data Science (TReNDS) Georgia State University, Georgia Institute of Technology, Emory University Atlanta Georgia USA; ^2^ State Key Laboratory of Cognitive Neuroscience and Learning Beijing Normal University Beijing China; ^3^ Department of Electrical and Computer Engineering University of Maryland Baltimore Maryland USA

**Keywords:** AD, multimodal fusion, multiple fMRI networks, overlapping fMRI, parahippocampus, parallel multilink jICA, posterior cingulate, thalamus

## Abstract

In this article, we focus on estimating the joint relationship between structural magnetic resonance imaging (sMRI) gray matter (GM), and multiple functional MRI (fMRI) intrinsic connectivity networks (ICNs). To achieve this, we propose a multilink joint independent component analysis (ml‐jICA) method using the same core algorithm as jICA. To relax the jICA assumption, we propose another extension called parallel multilink jICA (pml‐jICA) that allows for a more balanced weight distribution over ml‐jICA/jICA. We assume a shared mixing matrix for both the sMRI and fMRI modalities, while allowing for different mixing matrices linking the sMRI data to the different ICNs. We introduce the model and then apply this approach to study the differences in resting fMRI and sMRI data from patients with Alzheimer's disease (AD) versus controls. The results of the pml‐jICA yield significant differences with large effect sizes that include regions in overlapping portions of default mode network, and also hippocampus and thalamus. Importantly, we identify two joint components with partially overlapping regions which show opposite effects for AD versus controls, but were able to be separated due to being linked to distinct functional and structural patterns. This highlights the unique strength of our approach and multimodal fusion approaches generally in revealing potentially biomarkers of brain disorders that would likely be missed by a unimodal approach. These results represent the first work linking multiple fMRI ICNs to GM components within a multimodal data fusion model and challenges the typical view that brain structure is more sensitive to AD than fMRI.

## Graphical abstract

In this article, we proposed a multimodal fusion approach for joint analysis between multiple rest functional magnetic resonance imaging (fMRI) networks and MRI gray matter using multilink joint independent component analysis (jICA). There are several novel aspects of this study including joint relationship between multiple rest fMRI networks and gray matter, alternating learning of jICA parameters, etc. Results show significant differences in joint coupling between Alzheimer's disease and controls.

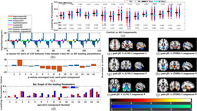

## 1 INTRODUCTION

In the context of the brain imaging studies, there are different imaging techniques to acquire neuroimaging data for visualizing neural activity, improving understanding of brain mechanisms, and identifying biomarkers‐especially for mental disorders. These techniques have provided remarkable new insights into human brain mapping to investigate the structural and functional changes of the human brain during transition from healthy aging to psychiatric disease. A large number of studies collect multiple modality data such as structural magnetic resonance imaging (sMRI), functional magnetic resonance imaging (fMRI), electroencephalography (EEG), diffusion tensor imaging (DTI), magnetoencephalography (MEG), and other types of data from the same individual. Among them, resting‐state fMRI (rs‐fMRI) has been progressively applied to functional mapping. It is increasingly evident that changing functional activity is a reliable indicator of brain disorders for early diagnosis and treatments (Li et al., 2020; Whitfield‐Gabrieli et al., 2009). Lin et al. (2010) presented a semi‐blind spatial ICA approach to improve ICA performance in fMRI analysis using spatial constraints. Independent component analysis (ICA)‐based mapping has been widely used, as no a prior information is required, and thus can be used to explore the entire dataset. Badhwar et al. (2017) studied multiple regions of rs‐fMRI and observed changes in functional activity with Alzheimer's disease (AD). Zhang et al. found that the dysfunction of salience network (SAN) and default mode network (DMN) may lead to discrepancy in attention in mild cognitive impairment (MCI) and AD patients (Zhang et al., 2018). Zheng et al. also studied rs‐fMRI and showed that AD group had lower functional connectivity than the healthy control (HC) especially within DMN, visual network (VN), and sensorimotor network (SMN) (Zheng et al., 2017). Only focus on single modality provides insubstantial information which may lead to incomplete inference than multimodal analysis. In addition to the modality‐based analysis, study of brain networks for biomarker development is becoming increasingly common. Many approaches have concentrated on a single network of the brain instead of multiple networks to identify indicator of brain disorders in neurological and psychiatric conditions, such as AD and schizophrenia (Chaovalitwongse et al., 2017; Du, Pearlson, et al., 2016; Toussaint et al., 2014), which might fail to uncover more comprehensive descriptions of altered brain connectivity than multiple networks. Therefore, the goal of this study is to fuse multiple networks and multiple modality data per subject to take maximal advantage of the join‐information of the existing data.

Two categories of approaches to capture joint information from multiple data sets include univariate approaches (e.g., correlation) and multivariate approaches (e.g., ICA). Multivariate approaches focus on interrelated patterns rather than unrelated points. They can estimate all the variables jointly by working with patterns instead of just paired relationships (Calhoun et al., 2009; Handwerker et al., 2012; Hardoon et al., 2009; Le Floch et al., 2012; Liu & Calhoun, 2014; Purcell et al., 2009; Vounou et al., 2010). Multivariate approaches can also discover hidden factors (sources or features) that underlie sets of random variables, measurements, or signals. ICA, as one widely used approach, assumes that the input mixed signal is a linear mixture of the sources, which are statistically independent and (mostly) non‐Gaussian. It performs independent source separation and noise component removal simultaneously (Du, Allen, et al., 2016). To estimate independent sources using ICA, statistical information higher than second order is needed. A variety of approaches for improving ICA algorithms have already been developed. The popular ICA algorithms that use nonlinear functions to generate higher‐order statistics can be linked to maximum likelihood estimation such as Infomax (Bell & Sejnowski, 1995; Lee et al., 1999), FastICA (Hyvarinen & Oja, 1997). Another algorithm named joint approximate diagonalization of eigenmatrices (JADE) explicitly computes fourth‐order cumulants of whitened signals (Cardoso & Souloumiac, 1993). Although both FastICA and Infomax are robust, both make some assumptions about the source distributions. They work well for symmetric distributions and are less accurate for skewed or multimodal distributions and for sources, which are close to Gaussian (Bach & Jordan, 2002; Boscolo et al., 2001; Calhoun et al., 2009; Du, Levin‐Schwartz, et al., 2016; Fu et al., 2014; Koldovský et al., 2006; Li & Adali, 2010). Both algorithms require the nonlinearities to tie the form of source distribution according to a given optimality condition. There are several adaptation strategies which have been developed to relax this requirement. Koldovský et al. introduced a generalized Gaussian density model (Koldovský et al., 2006). Other flexible adaptation methods include entropy bound minimization (EBM) (Lin et al., 2010), non‐parametric ICA (Boscolo et al., 2001), kernel ICA (Bach & Jordan, 2002) and other more recent approaches such as those based on entropy rate minimization that make use of both higher‐order statistics and sample dependence (Du, Levin‐Schwartz, et al., 2016; Fu et al., 2014).

Every imaging technique provides unique, filtered and interrelated information of brain's structural and functional organization (Calhoun & Sui, 2016; Sui et al., 2012). Combining modalities can help to provide a more complete understanding of the human brain and its disorder as compared to unimodal studies (Calhoun & Sui, 2016; Sendi et al., 2021). Utilizing multiple modality can leverage the joint or complementary information between them. AD is known to impact both function and structure, but the bulk of the focus has been on structural MRI (rather than functional MRI) as this tends to show higher sensitivity and predictive accuracy. However, there has been little work focused on the identification of functional and anatomical interactions among different brain regions (Bozzali et al., 2011; Brier et al., 2014). Luo et al. (2020) showed structural–functional relationship using a dataset of over 1500 individuals, but they used a two‐step process, first performing separate, rather than joint, analysis. Sendi et al. (2021) studied the progression from normal brain to very mild AD (vmAD) using separate multimodal analysis. Jointly analysis multiple types of imaging data from same individual can be particularly useful in this regard. Qi et al. (2022) developed a three‐way parallel group ICA fusion method to discriminate schizophrenia and controls. Duan et al. (2020) proposed a multimodal fusion approach called aNy‐way ICA, which is capable of detecting multiple linked sources over any number of modalities without involving orthogonality constrains on sources. Joint ICA (jICA) is another useful data fusion approach which allows us to take advantage of the cross‐information among different modalities (Moosmann et al., 2008).

However, jICA has a number of limitations. First, it assumes that different modalities come from the same distribution. Second, typically the rich fMRI data are reduced to a single map per subject, for example, the amplitude of low frequency fluctuation (ALFF) (Hare et al., 2017; Turner et al., 2012; Zheng et al., 2021) or a single intrinsic network like default mode. In this study, our goal was to fuse gray matter (GM) and multiple rest fMRI networks, called intrinsic connectivity networks (ICNs). The GM includes volume values following preprocessing and Jacobian modulation. As an initial approach, we analyze this fusion using the same core algorithm as jICA. This enables us to examine multiple fMRI networks associated with the sMRI data. However, GM maps have a very different distribution from ICNs derived from ICA, which are already maximally independent (Yuhui et al., 2020). As we show, this can then lead to a local minima problem or poor optimization in the jICA. To address these limitations, we propose an extension called parallel multilink jICA (pml‐jICA) to fuse GM with multiple fMRI networks. We compared the proposed parallel approach with the use of “regular” jICA to link the GM maps with multiple fMRI networks, called multilink jICA (ml‐jICA). As we show, pml‐jICA provides a more balanced weight distribution over ml‐jICA/jICA and it can extract relationship between brain function and brain structure from multiple rest fMRI networks. The illustrated advantages of pml‐jICA over ml‐jICA also indicate improvements over jICA due to our use of the same jICA core algorithm for ml‐jICA. There are multiple novel aspects of this article: (1) we study the joint relationship between resting function and structure, (2) alternating learning of the jICA parameters between modalities (rather than concatenated learning) to ensure balance across modalities and can scale to more modalities, (3) inclusion of multiple ICA component maps (resting networks) as multiple rows for each subject each linked to the same GM map, and (4), analysis and visualization of multiple loading sets for each subject. Results show significant differences between specific linked sMRI/fMRI ICNs. The proposed pml‐jICA approach reveals some of the expected areas including hippocampus, opposite overlap in DMN, and some subcortical areas, which are notable network for AD progression from healthy to patients.

## 2 MATERIALS AND METHODS

### 2.1 Imaging data acquisition

In this article, the experimental data we used are from the longitudinal Open Access Series of Imaging Studies (OASIS‐3). Data were collected from several ongoing studies in the Washington University Knight Alzheimer Disease Research Center over the course of 15 years (LaMontagne et al., 2019). The dataset is a compilation of MRI and PET imaging and related clinical data for 1098 participants. It has over 2000 MR sessions including multiple structural and functional sequences. We used sMRI and resting state BOLD sequences for our analysis. In order to evaluate neural activity at rest, participants were asked to lay quietly with their eyes open while two 6 min resting state BOLD sequences were collected. A total of 2165 MR sessions (236 1.5T scanner and 1929 3.0T scanner) for sMRI with T1w scan type, 1691 MR sessions (2 1.5T scanner and 1689 3.0T scanner) with BOLD‐resting state scan type, and other varieties of scan types were included in OASIS‐3 dataset. T1w images were segmented via statistical parameter mapping (SPM12, http://www.fil.ion.ucl.ac.uk/spm/). For each participant, the imaging data, demographics information, and clinical dementia rating (CDR) scale were used at any stage of cognitive functionality. According to the scale, all participant must have CDR ≤ 1 at the time of the clinical core assessment. If one participant gained CDR 2, they were no longer eligible for the study. Among 1098 participants, 850 participants entered with cognitively normal adults; 605 of those remained normal while 245 converted to cognitive impairment at various stages with ages ranging from 42 to 95 years (LaMontagne et al., 2019). The CDR scale of the additional 248 participants was greater than zero.

### 2.2 Quality control and data preprocessing

The fMRI data were preprocessed using SPM12. Rigid body motion correction followed by slice‐timing correction was performed to correct subject head motion and to account for timing difference in slice acquisition. The fMRI data were then subsequently normalized into the standards Montreal Neurological Institute (MNI) space and resliced to 3 mm × 3 mm × 3 mm isotropic voxels. The resliced images were further smoothed using a Gaussian kernel with a full width at half maximum (FWHM = 6 mm). Next, we analyzed the data to identify 53 ICNs using the NeuroMark pipeline, which is a fully automated spatially constrained ICA approach (Yuhui et al., 2020). The Neuromark_fMRI_1.0 component templates were used as spatial priors. The sMRI data were segmented and spatial normalized using the SPM unified segmentation approach, following by Gaussian smoothing with a FWHM = 6 mm kernel. For quality control, the GM images have been divided into a random number of groups. After that, quality control was performed by spatially correlating each individual GM image with the group average images across all subjects and flagged scans with a correlation below 0.95. This quality control process refined the dataset and reduced the number of subjects from 1098 into 784, include 648 HCs and 136 individuals with AD. We analyzed a final set of randomly selected 130 HCs and 130 AD subjects from the refined dataset in which the control and patient sample sizes were the same. The HCs are categorized as cognitively normal with CDR equals 0 and the ADs are categorized as AD dementia with CDR ≥ 0.5. The final dataset was further preprocessed by reslicing and Gaussian smoothing to match the voxel sizes (though this is not strictly a requirement of the approach). Finally, we prepared the two different types of data, GM and fMRI, for joint analysis by normalizing to unit variance across all subjects within each modality. The demographic and clinical information of the refined experimental data are summarized in Table 1.

**TABLE 1:** Demographic and clinical information.

	*N*	130
ADs	Gender (M/F)	54/76
	Age (entry)	42.45–94.93
	CDR	0–0
	SOB	0.0–0.5
HCs	Gender (M/F)	68/62
	Age (entry)	50.29–95.57
	CDR	0.5–2.0
	SOB	0.5–12.0

Abbreviations: AD, Alzheimers disease; CDR, clinical dementia rating; HC, healthy control; SOB, sum of boxes.

### 2.3 Joint ICA and multilink jICA (ml‐jICA)

Consider a set of two equations for two sets of group data (there can be more than two, but for simplicity, we first consider two modalities):

(1) $$x^{\left(1\right)}=As^{\left(1\right)}\;\text{and}\;x^{\left(2\right)}=As^{\left(2\right)}$$

where $x\left[m\right]$ and $s\left[m\right]$ are the random vectors for modality m, and **A** is the mixing matrix. For a unimodal system, we can write the likelihood functions $p\left(X^{\left(1\right)};W\right)$ and $p\left(X^{\left(2\right)};W\right)$, considered as functions of W, where $W=A^{-1}$, ignoring scaling and permutation ambiguities. Therefore, we maximize these two likelihood functions in a separate ICA analysis. This would give two unmixing matrices for each modality. That is, we take our estimator separately as, ${\hat{W}}_{1}=\arg\max_{W_{1}}\log\left(X^{\left(1\right)};W_{1}\right)$ and ${\hat{W}}_{2}=\arg\max_{W_{2}}\log\left(X^{\left(2\right)};W_{2}\right)$. We utilize a data fusion approach to combine the two sets of optimal unmixing coefficients in the jICA.

**FIGURE 1:**
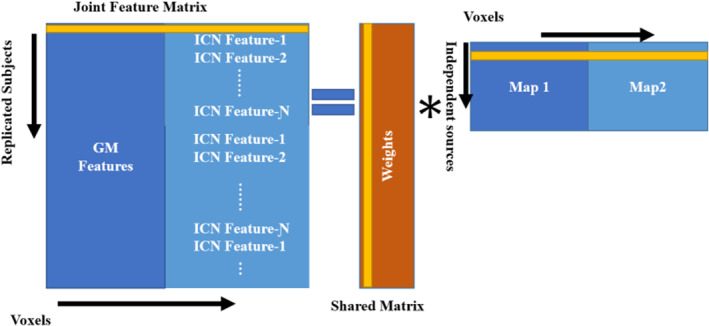
Gray matter (GM) features and intrinsic connectivity networks (ICN) features are stacked side by side to create a joint matrix. The number of ICN (Ɲ) is stacked vertically. The joint matrix is then modeled as a spatially independent joint sources images which share common mixing parameters.

In ml‐jICA, we use the same core algorithm, but it is applied to the multiple fMRI networks through a novel approach. A conceptual model of the ml‐jICA is represented in Figure 1, which demonstrates the configuration of input feature matrix and shared mixing matrix with joint sources. Maps 1 and 2 represent the GM and rest fMRI network part of the joint components, respectively. We are mainly interested in linking a targeted linear combination for multiple fMRI networks with the GM data. While the ICNs are maximally independent within the fMRI data, they have not been optimized for the joint fusion with GM data. We use vertical stacking of the subject‐specific component maps of the ICNs instead of horizontal stacking because our goal is to fuse structure and function without losing information by selecting a single ICN or reducing fMRI to a single image via ALFF or some other approaches (Hare et al., 2017; Turner et al., 2012; Zheng et al., 2021). We do not expect the networks to covary the same way, and there are multiple ICN, so horizontal stacking was not the right solution there.

**FIGURE 2:**
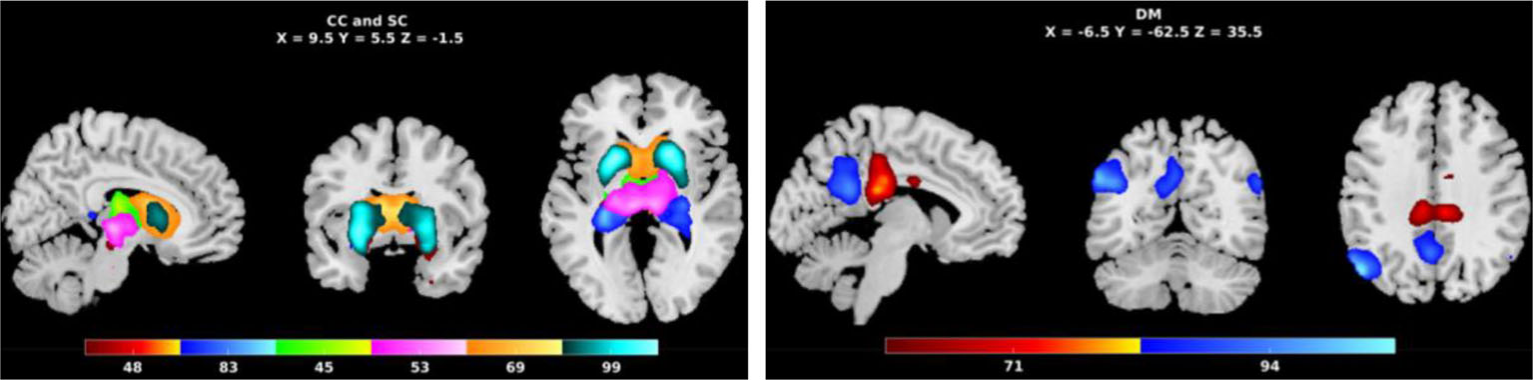
Eight‐rest fMRI networks; CC, cognitive control domain (hippocampus [48, 83]); SC, sub‐cortical domain (thalamus (Li et al., 2007), hypothalamus (Rao et al., 2022), caudate [69, 99]); DM, default‐mode domain (posterior cingulate [71, 94]).

In this study, to minimize memory and computation requirements, and based on prior work suggesting hippocampus and default mode as key regions of interest for AD, we investigated eight‐rest fMRI networks including two ICNs which overlapped with hippocampus (48, 83) from the cognitive control domain, two ICNs including caudate nucleus (69, 99), thalamus (Li et al., 2007), and hypothalamus (Rao et al., 2022) from the subcortical domain, and two posterior cingulate networks (71, 94) from the DMN domain (Yuhui et al., 2020). These are shown in Figure 2. The linear combination of the ICNs for a subject can be defined by the following equation,

$$x_{1}^{{\mathrm{ICN}}_{k}}=\sum_{l=1}^{ɳ}\beta_{k,l}{\mathrm{ICN}}_{l},$$

where $x_{1}^{{\mathrm{ICN}}_{k}}$ is a linear combination of the ICN for subject‐1, which is replicated k times (k=1,2,…,8); $\beta_{k,l}$ represents scaled uniformly distributed random numbers, where $l=1,2,\ldots ɳ\;\left(ɳ=8\right)$ for each ICN. This is applied at the data preprocessing stage before feeding into the ml‐jICA and pml‐jICA algorithms. Principal component analysis (PCA) is used to reduce the dimensionality of the joint multimodal input data, GM and linearly combined ICNs. Every subject‐specific component map of fMRI network is stacked side by side with GM. In this representation, the GM feature of each subject is replicated for the multiple rest fMRI networks.

A generative model of the given datasets, $X^{G}$ and $X^{\mathrm{ICN}}$, can be written as, $x^{G}=As^{G}$ and $x^{\mathrm{ICN}}=As^{\mathrm{ICN}}$, where, for the case of two modalities and two subjects, $x^{G}={\left[x_{1}^{G}\;x_{2}^{G}\right]}^{T}$ is the mixed data for the GM modality, and $x^{\mathrm{ICN}}={\left[x_{1}^{\mathrm{ICN}}\;x_{2}^{\mathrm{ICN}}\right]}^{T}$ is the mixed data for the ICN (rest‐fMRI modality); $A=\left[\begin{array}{cc} a_{11} & a_{12}\\ a_{21} & a_{22} \end{array}\right]$ is the shared mixing coefficient matrix, and $s^{G}$ and $s^{\mathrm{ICN}}$ are the GM and ICN sources, respectively. We can write observation vector for each subject as a single expression, $x_{i}=\left[x_{i}^{G}\;x_{i}^{\mathrm{ICN}}\right]$, similarly for the source vector, $s_{i}=\left[s_{i}^{G}\;s_{i}^{\mathrm{ICN}}\right]$; The resulting joint equation for subject i is then $x_{i}=As_{i}$. The resulting joint equation for all of the subjects can be written in this matrix form,

(2) X=AS

After concatenating the two datasets to form $X^{J}$, the likelihood is written as,

$$L\left(W\right)=\prod_{n=1}^{N}\prod_{v=1}^{V}p_{n}^{J}\left(u_{v}^{J}\;\right),\text{where}\;u^{J}=Wx^{J}$$

Each entry in the vectors $u^{J}$ and $x^{J}$ are replaced by the observation for each sample n=1,…,K,…,N as rows of matrices $U^{J}$ and $X^{J}$. If the two sets of group data have dimensionality $N\times V_{1}$ and $N\times V_{2}$, then the joint likelihood can be written as,

$$L\left(W\right)=\prod_{n=1}^{N}\left(\prod_{v_{1}=1}^{V_{1}}p_{n}^{G}\left(u_{v_{1}}^{G}\right)\prod_{v_{2}=1}^{V_{2}}p_{n}^{\mathrm{ICN}}\left(u_{v_{2}}^{\mathrm{ICN}}\right)\right),$$

for the maximum likelihood (ML) estimator based jICA.

In this study, we utilized Infomax algorithm (Bell & Sejnowski, 1995) to estimate the common unmixing matrix for the joint analysis. The application of Infomax to source separation consists in maximizing an output entropy. It is implemented by maximizing the following ML score function with a fixed (sigmoid/tanh) nonlinear function:

(3) $$\varphi_{I}\left(W\right)≝H\left(g\left(\mathrm{Wx}\right)\right)$$

Infomax is directly linked to maximum likelihood and the nonlinearity used is a good match for super‐Gaussian sources, and hence for fMRI/MRI analysis as well ( Adali et al., 2014). For the data fusion analysis, joint data matrix is created as (we consider two modalities for simplicity), $X=\left[X^{\left(1\right)},X^{\left(2\right)}\right]$, placing one beside the other (see Figure 1), similarly, the source matrix is created as $S=\left[S^{\left(1\right)},S^{\left(2\right)}\right]$, in which each of the rows represents the number of voxels in two images, components of the two modalities for each subject. Then, the Equation (3) can be modified as,

(4) $$\varphi_{I}\left(W\right)≝H\left(g\left(\mathrm{WX}\right)\right)$$

The model could jointly identify paired components by linking with multiple rest fMRI networks and optimize the decomposition for a variable of interest.

### 2.4 Parallel multilink jICA

The original jICA formulation assumes that the joint sources with the two data types (GM and fMRI) modulate the same way across N subjects, that is, the sources have a common modulation profile across subjects as well as statistical independence among the joint sources. Assuming inter‐subject variations of multimodal sources to be the same for different modalities is a strong constraint. While statistical independence may be a feasible assumption, the jICA algorithms may not be able to accomplish both of the constraints at the same time. It may end up achieving a tradeoff solution between the two constraints (Correa et al., 2008). In addition, the jICA model requires contributions from both modalities to be similar, so that one modality does not dominate the other in the estimation of the maximally independent components. If the two datasets have different ranges, the datasets need to be normalized in the preprocessing stage.

In this experiment, we used GM and subject‐specific spatial maps of the ICNs, where ICNs are already maximally independent, that is, output of ICA, which have very different distributions. Therefore, the jICA and/or ml‐jICA models are in conflict with the i.i.d. assumptions. To address this, and generalize jICA, we proposed an adaptation approach for fusing GM and multiple rest fMRI networks by parallel optimization.

**FIGURE 3:**
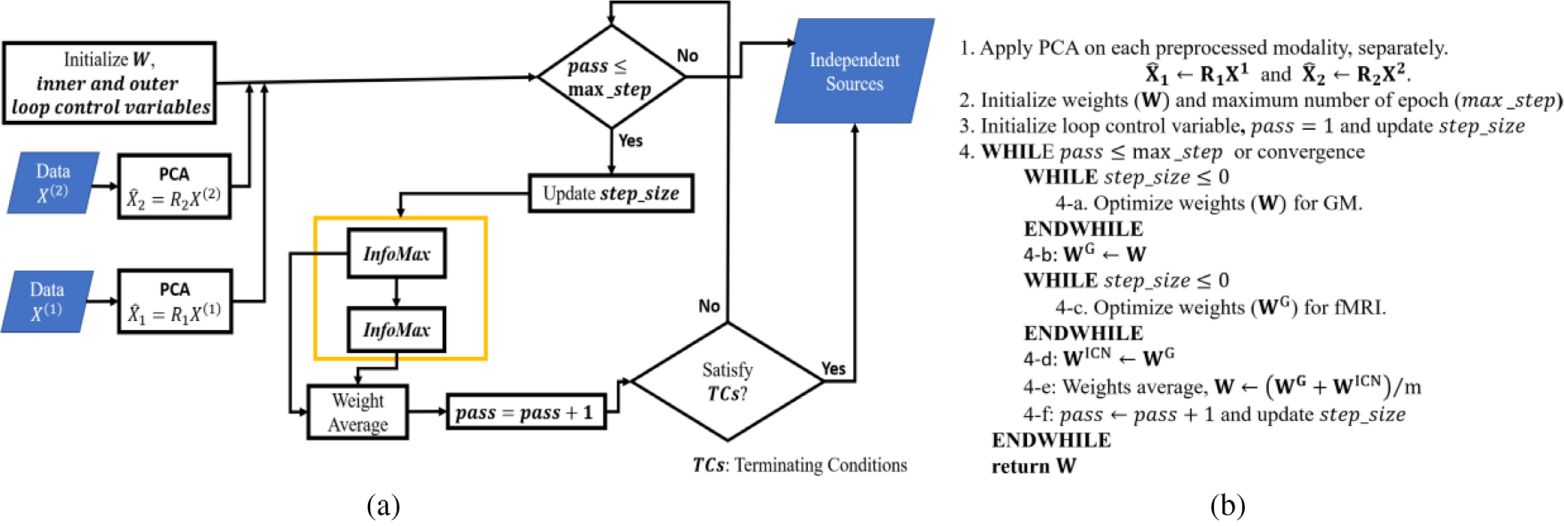
(a) Diagrammatic representation of the proposed parallel multilink jICA (pml‐jICA) approach; (b) pseudo‐code for the optimization approach.

In the parallel optimization approach, the weights of each modality are optimized separately with different number of iterations for each pass. The weights are initialized for each pass by taking average after the number of iterations. Finally, the mixing loading matrix is calculated by projecting the final average weights on the joint feature matrix, which is calculated from the joint data matrix. The overall optimization approach is illustrated in Figure 3. $X^{1}$ and $X^{2}$ are datamatrix for GM and fMRI with size $N\times V_{1}$ and $N\times V_{2}$, respectively, where N is the number of subjects and $V_{1}$ and $V_{2}$ are the number of voxels, and m represents the number of modalities. Each datamatrix was normalized by mean across subjects and then scaled within each modality. Before weight optimization, principal component analysis (PCA) is used to reduce dimensionality of the data, and decorrelate the data using second‐order statistics, where whitening matrices, denoted as $R_{1}$ and $R_{2}$ (Figure 3), are same. After sphering the data, the initial weights are optimized iteratively by maximizing an output entropy until max_ste ***p*** or satisfy the terminating conditions. The iteration number is controlled by step_size. After every pass, the iterative optimized weights, $W^{G}$ and $W^{\mathrm{ICN}}$ **,** are updated for GM and fMRI, respectively.

**FIGURE 4:**
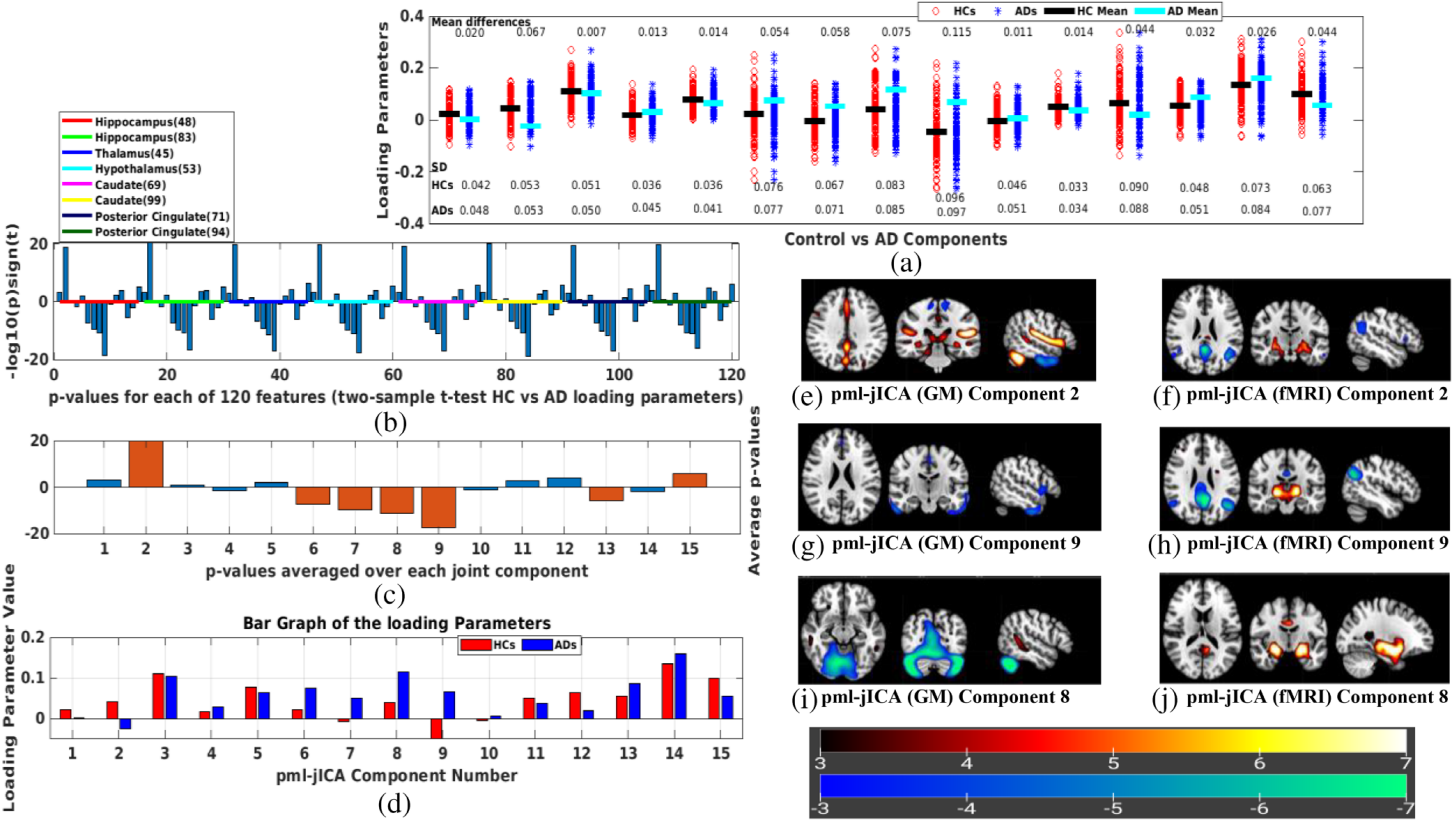
(a) Demonstrating loading parameters between control versus Alzheimers disease (AD) components; (b) *t‐*test between controls (CNs) and ADs; (c) Average *p*‐values of the rest functional magnetic resonance imaging (fMRI) networks; (d) Bar graph of the loading parameters between CNs and ADs. (e)–(j) represent joint source map (pml‐jICA) for Components 2, 9, and 8, respectively; Splitting the overlapping fMRI default mode network (DMN) into two components ([f] and [h]); (e) and (f) show gray matter changes in posterior cingulate regions near the fMRI default mode posterior cingulate peak. (e)–(h) represents coupling between multiple networks including precuneus, posterior cingulate, hippocampus, and some subcortical regions. (i) and (j) represent joint relationship between cerebellar area of GM, and parahippocampal gyrus (PHG) and some regions of subcortical domain (SC) in the fMRI.

**FIGURE 5:**
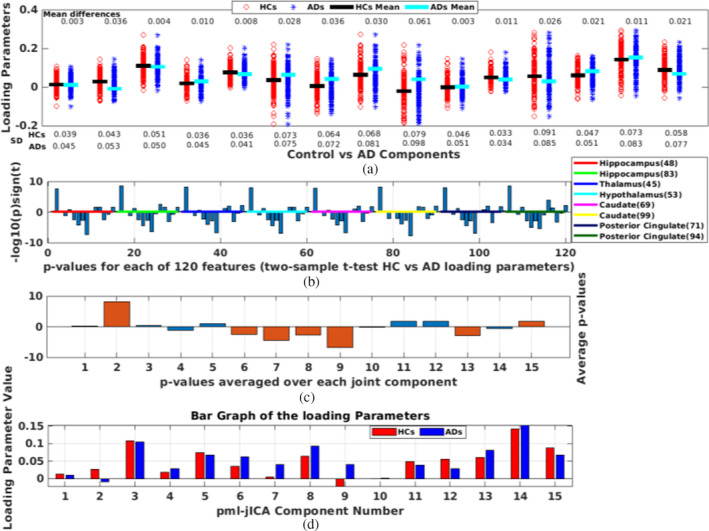
Resulting loading parameters and two sample *t*‐test results after regressing out age. Overall pattern results are same as before (Figure 4). The results represent strong ability to differentiate between the two groups.

After estimating 15 components, we analyze group difference between HCs) and ADs using two‐sample *t*‐test followed by false discovery rate (FDR) correction for multiple comparisons. Since we have included eight ICNs and extracted 15 components, we have 120 features to compare. We compared each of these features and showed the results in Figure 4. We repeated the same analysis after regressing out the effect of age before performing the t‐test, these results are presented in Figure 5.

## 3 RESULTS

In this section, we present results and performance analysis for ml‐jICA and pml‐jICA. Our example shows result from GM and eight‐rest fMRI networks from each subject where 130 HCs and 130 ADs subjects are investigated in this experiment. We optimized the data matrix differently for ml‐jICA and pml‐jICA. The data matrix is created separately for the rest fMRI networks and GM, respectively, prior to either concatenation or joint optimization. Each subject has multiple rows corresponding to each of the eight‐rest fMRI networks and the sMRI data matrix is replicated to provide corresponding columns. The number of rows for both fMRI and GM is 2080 (eight times the number of subjects) for 260 subjects. For the pml‐jICA, the fMRI and GM data matrices are analyzed in a slow optimized fashion. First, the weights of the fMRI and GM are optimized with one iteration in the first pass, then optimized with four iterations from second to 20th pass, then optimized with eight iterations from 21 to 40th pass; increasing in this way the weights are optimized slowly to reach convergence. For every pass, weights are averaged before the next pass.

We estimated 15 joint components. The number of components is a free parameter, which can be either determined from empirical experience or estimated using information theoretic approaches (Beckmann & Smith, 2004; Li et al., 2007). The chosen component number is approximately twice of the number of networks since we were interested in a more fine‐grained fusion. Based on empirical results, this number provided a good option to separate signals and noise into different independent sources. Results from the estimated components showed several interesting patterns, and also surprisingly large group differences between AD and HC for some components. The loading parameters separated by group are shown in Figure 4a for the pml‐jICA. The red circles and blue asterisks represent loading parameters for controls and ADs, respectively. We found several components that demonstrated highly significantly different loading parameters in patients and controls. The black line segment is the mean value of the control and the cyan line segment is the mean value of the patient. The mean difference (min_diff) between two groups show us how much they differ in patients and controls. The standard deviation (SD) of the loading parameters tells us how the weights vary across subjects.

To quantify correlations among the fMRI networks at rest and to find most significant components, we perform two sample *t*‐tests between controls versus AD for each rest fMRI network with significance level 0.001. From eight fMRI networks and 15 joint components, we estimated 120 *p*‐values. The estimated *p*‐values (−log10 (p)*sign (T)) are shown in Figure 4b. The colored lines separate the *p*‐values for each fMRI ICNs. We observe generally similar patterns among the different networks/ICNs, although as we show later, there is also some interesting variation. After taking component‐wise average of all networks, we found several significant components based on the threshold for statistical significance, which was set at corrected <0.001. The *p*‐values for the significantly differing components 2, 6, 7, 8, 9, 13, and 15 are shown in Figure 4c. Components are marked with dark orange color have *p*‐values below 0.001. The average loading parameter values for the controls and patients are shown in Figure 4d. These show us both the degree to which the component is expressed in each group, and also whether it is positively or negatively expressed. Components 2 and 9 represent the most significant difference between controls and ADs.

The seven joint source maps that showed significant group differences were thresholded separately for GM and fMRI. The resulting voxels show the ones that contribute strongly to these components either positively or negatively. Multiple networks were identified for GM and rest fMRI by the proposed approach. Five of these components (Calhoun et al., 2009; Chaovalitwongse et al., 2017; Du, Pearlson, et al., 2016; Toussaint et al., 2014; Zheng et al., 2017) contained areas where joint source values were higher in ADs than controls; two components (2 and 15) had active areas where joint source values were greater in controls than patients (Figure 4c). We summarized important activated brain regions of the seven spatial patterns having volumes greater than 0.4 cm^3^. First, we described here the most interesting patterns. The top two largest differences between diagnostic groups were found in Components 2 and 9. The Component 2 (min_diff=0.067,p−value=3.9e−20) and Component 9 (min_diff=0.115,p−value=1.82e−17) formed a consistent spatial grouping, where Component 2 indicates greater volume in controls than ADs and Component 9 indicates lower volume in controls than ADs (Figure 4c). If we notice the two components, we see that they have slightly different GM maps, and both subtractive in AD; but splitting the fMRI DMN into two different components, one is subtractive and another one is additive in AD. The most interesting finding here is that our joint analysis (pml‐jICA) separates overlapping regions of the fMRI DMN into two different ICs from the input data, which is illustrated in Figure 4e–h. Another interesting finding is in the Component 8, which represents joint relationship between cerebellar area of GM and parahippocampal gyrus (PHG) and subcortical domain (SC) of the brain in fMRI. Figure 4i,j depicts spatial pattern and their activated brain regions for Component 8. In Component 6, the activated voxels of the GM are mostly in sensorimotor, cortical, and cerebellar regions, where sensorimotor and cortical regions are lower in AD, but cerebellar regions are increased in AD; most regions in the fMRI are subcortical, so the shift to cerebellum from cortical regions is linked to thalamic and subcortical functional increases in AD. In Component 7, caudate reduces and thalamus increases in the fMRI; precentral and frontal areas increase in AD but lower in DMN. In GM of the Component 13, the cerebellum increases and parahippocampal decreases in AD. In Component 15, cingulate regions are reduced structurally in AD and parahippocampal decreases functionally in AD. Most of the red regions of fMRI seem to be ventricles, which are less interesting. A brief summary of all significant components is presented in the supplementary materials. To see the robustness of this method in differentiating the two groups, we regressed out the variance associated with the covariate, and recompute the two‐sample *t*‐test. The results also shown in Figure 5. The overall pattern results are similar even after regressing out the age. Age of course is a predictor of AD by itself, but even after removing age, our method shows a strong ability to differentiate between controls and AD subjects, and importantly, age alone does not lead to the observed patterns described in the analysis.

## 4 MODEL PERFORMANCE AND COMPARISON

**FIGURE 6:**
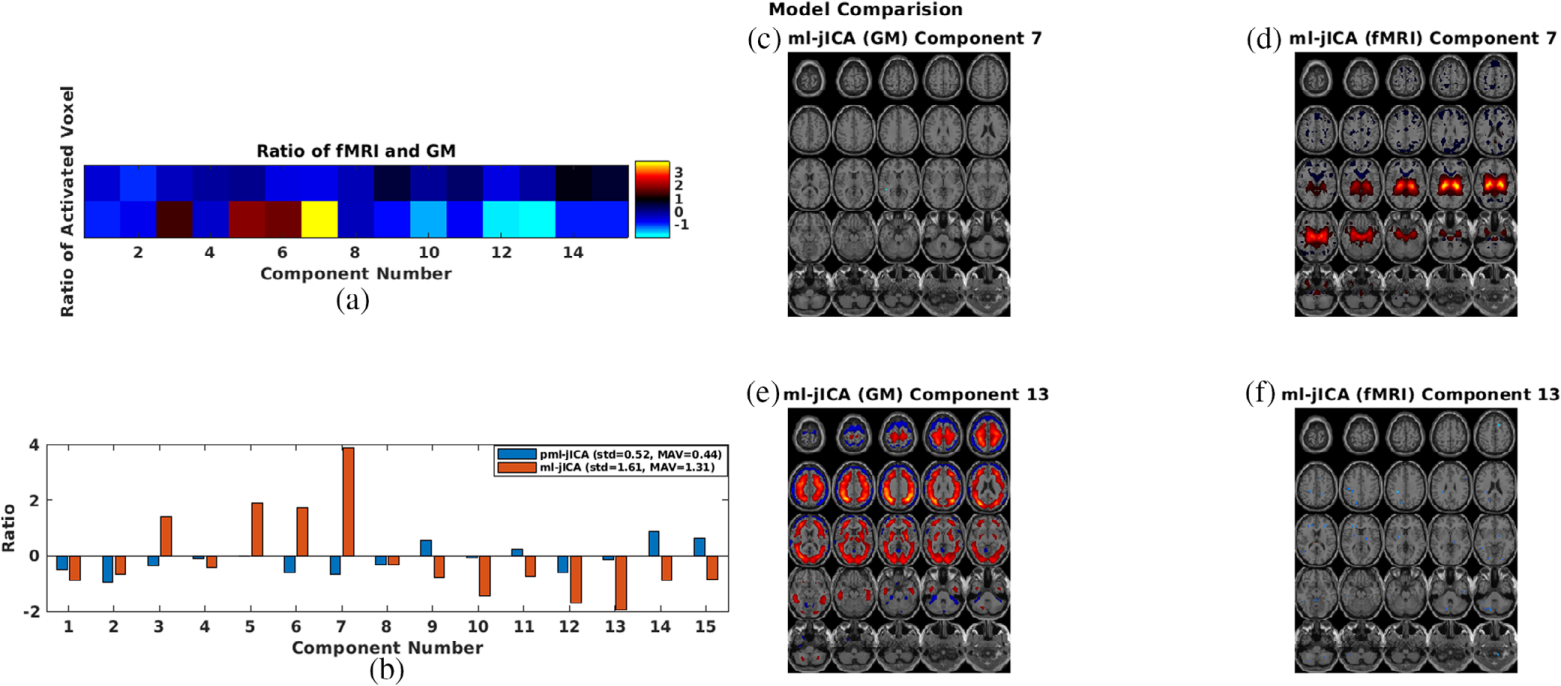
Weight distribution among modalities for model comparison. The color bar indicates the color mapping for the source maps. The ratio (Figure 6a,b) between gray matter (GM) and functional magnetic resonance imaging (fMRI) indicate imbalance pattern of weight distribution in the multilink joint independent component analysis (ml‐jICA) over parallel multilink jICA (pml‐jICA). Figure 6c–f illustrate the weight contribution differences.

Results from our comparison of ml‐jICA and pml‐jICA in terms of weight distribution are demonstrated in Figure 6. This figure illustrates the weight distribution between modalities. Our expectation was that ml‐jICA would be more likely to fall into a “modality specific” mode due to the different distributions of GM and ICNs. We calculated the ratio of the number of strongly contributing voxels (activated voxels after thresholding) for each component between fMRI and GM, shown in Figure 6a,b. One of our observations of ml‐jICA was that the learning tended to converge to either fMRI only sources, or joint sources (see Figure 6c–f for examples of this). This is likely an effect of pooling together maximally independent courses and more Gaussian‐like features (in the GM maps) for the optimization, and mixing them together in a single optimization. This behavior can also be quantified by evaluating the standard deviation of the ratio, mean absolute value (MAV), and the image (Figure 6b). Our pml‐jICA approach was developed to learn the weights from either GM or fMRI, followed by initialization and optimization of the other modality and alternating until convergence. Visual results (Figure 6a,b) showed a more balanced result where all the components are contributed to by both modalities. Results from this plot show more extreme values for the ml‐jICA, and for pml‐jICA indicates that weights are more evenly contributed to by fMRI and GM than ml‐jICA. Our proposed algorithm provides a solution which balances the contributions of two modalities and allows for joint estimation from multimodal data with vastly different distributions. The source maps of the two most roughly distributed components (Components 7 and 13) are shown here as an illustration (Figure 6c–f).

## 5 MULTILINK NETWORK CONTRIBUTION

**FIGURE 7:**
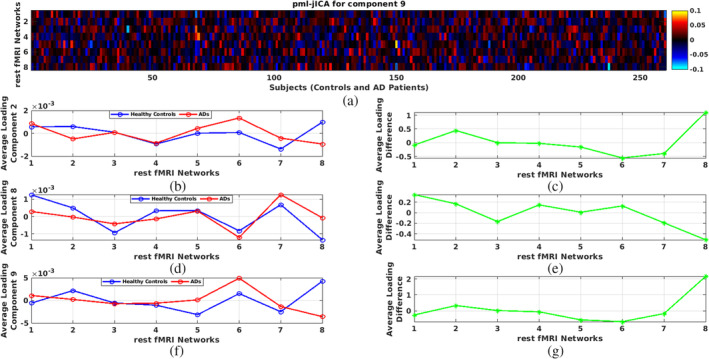
Multilink network contribution in the 2, 8, and 9 independent components; (a) representing variability in linking between different networks across subjects in the Component 9; (b)–(g) demonstrating network contribution in the Components 2, 8, and 9 as a representative of the 15 estimated components.

While the overall patterns of multiple networks within subjects were similar, there were also some important differences. We can see this in Figure 7, Figure 7a shows loading contribution relative to mean for each subject across eight‐rest fMRI networks. This highlights the variability that is present. To further unpack this, we can see in Figure 7b–g the relative contribution for each fMRI network for CNs and ADs separately and also the difference between CNs and ADs. The right image of Component 2 (Figure 7c) indicates that the posterior cingulate of DMN has greater representation in CNs than in ADs. Figure 7c,g tell us that the posterior cingulate component has the strongest contribution among all of the eight‐rest fMRI networks. The posterior cingulate cortex is a highly connected and metabolically active brain network. Our result highlight abnormal functional connectivity in the posterior cingulate network, which is consistent with, but significant expands, prior work (Lee et al., 2020; Leech et al., 2012; Mutlu et al., 2016; Yu et al., 2017; Zhang et al., 2017).

## 6 DISCUSSION

Our study examines the structural and functional changes between HCs and AD patients. We found interesting structural and functional pattern especially in precuneus, posterior cingulate, hippocampus, insula, superior frontal gyrus, medial and middle frontal gyrus, supplementary motor area, thalamus, putamen of the default mode, cognitive control, sub‐cortical regions, respectively, as well as cerebellar and sensorimotor regions of the brain; which are known to be significantly affected areas for AD (de Jong et al., 2008; Henneman et al., 2009; Koch et al., 2022; Lee et al., 2020; Leech et al., 2012; Mavroudis, 2019; Miao et al., 2011; Mutlu et al., 2016; Rao et al., 2022; Yu et al., 2017; Zhang et al., 2017; Zhong et al., 2014). The DMN plays an important role in functional brain structure. The PCC has been shown to have a significant causal relation with other nodes (Miao et al., 2011). Neuropathological atrophies are mostly distributed in posterior cortical regions, for example, precuneus, posterior cingulate, of the brain in the early stage of AD (Koch et al., 2022; Mutlu et al., 2016). In addition to the DMN, the hippocampus, the area in the cognitive control (CC) of the brain that is important for memory, may be potential biomarkers to predict the progression from MCI to AD (de Jong et al., 2008; Henneman et al., 2009; Rao et al., 2022). In addition, de Jong et al. (2008) found that the volumes of putamen and thalamus were significantly changed in patients diagnosed with probable AD.

Results for the highly significant components are shown in Figure 4e,f. In the fMRI part of the joint component, angular gyrus and some parts of the DMN show negative activation whereas hippocampus shows positive activation. In the GM part of the joint component, some parts of the DMN, thalamus, parahippocampus, and cerebellum show positive activation. We also see some portions of the posterior cingulate show both fMRI and GM changes. Besides, the superior temporal gyrus is additive and inferior temporal gyrus is subtractive. Some parts of the sensorimotor (precentral, paracentral lobule) are also subtractive. The loading directionality of this component is CNs>ADs (Figure 4c). So, the hippocampus represents functionally lower connectivity in AD group as well as the thalamus, parahippocampus, and cerebellar are structurally reduced in GM which consistent the previous studies (de Jong et al., 2008;Miao et al., 2011; Mutlu et al., 2016; Zhong et al., 2014). In Figure 4g,h, angular gyrus and some parts of DMN (especially posterior cingulate area) have negative activation whereas thalamus has positive activation in fMRI. From GM, we see that inferior temporal gyrus of the auditory area and supplementary motor area reduce volume in ADs than CNs.

If we compare Components 2 and 9 of pml‐jICA, we see that our proposed approach separates overlapping regions of the DMN into the two components, which is highlights an important benefit of the pml‐jICA. We observed that some portion of the DMN (especially precuneus) changes into another portion of the DMN (especially posterior cingulate) from Component 2 to Component 9, where one is additive and another one is subtractive, which implies that AD group has lower functional connectivity in the posterior cingulate area than the healthy group. This inference is also consistent with previous studies (Miao et al., 2011; Mutlu et al., 2016; Zhong et al., 2014).

In Component 8, precuneus (left), hippocampus, and putamen networks of the DMN, CC, and SC, respectively, are additive in the fMRI, whereas cerebellar network is subtractive in the GM (Figure 4i,j). This component shows coupling between cerebellar structurally and hippocampus and some subcortical areas of the brain functionally. The hippocampus and activated subcortical areas show higher connectivity in AD group whereas cerebellar reduces in AD group.

From Components 2, 8, and 9, we may infer that posterior cingulate, hippocampus, and some subcortical areas are the most important network for AD disease progression from healthy to AD patients. Our multilink network contribution analysis also tells us the same implication as the joint source analysis.

## 7 CONCLUSIONS

We presented two multimodal fusion approaches, called ml‐jICA and pml‐jICA, to examine the relationship between healthy and Alzheimer's individuals. The ml‐jICA uses the original jICA algorithm with a vertical stacking of multiple functional networks linked to the same GM networks. This is to avoid losing information due to fusing the two modalities. However, this still makes the same i.i.d assumption as jICA, which we show requires an additional modification due to the very different distribution of GM and ICNs. The main purpose of this study is to discover unique components from rest fMRI networks and GM maps, which share similar correspondence between subjects. This then enables to identify which discovered components best describe and differentiate between patient and control groups. There has been little work combining ICNs with GM data, and our results suggest this may ignore some information that can inform us about the impact of AD on the brain. In this initial work, we fused eight rest fMRI networks with gray matter. The main advantage of pml‐jICA approach is that it maximizes the joint likelihood function of multiple ICN or brain networks with GM provides a more complete and sensitive solution than one that does not utilize joint statistics. Also, the vertical stacking preserves more structural and functional information than horizontal stacking via ALFF or other approach. Our results represented advantages of the pml‐jICA over ml‐jICA in the applications of examining AD impairment. The results also suggest that posterior cingulate network is the most contributing network among the eight‐rest fMRI networks. In future, we will further optimize pml‐jICA to incorporate more ICNs in order to provide a more complete structure/function mapping across the brain.

## CONFLICT OF INTEREST STATEMENT

The authors declare no conflicts of interest.

## Supporting information


**Data S1.** Supporting Information.

## Data Availability

The data are used from the Open Access Series of Imaging Studies (OASIS‐3) and can be access via the following website “https://www.oasis-brains.org.”
