# Supplementary material for: Multimodal fusion of multiple rest fMRI networks and MRI gray matter via parallel multilink joint ICA reveals highly significant function/structure coupling in Alzheimer's disease
Source: Hum Brain Mapp. 2023 Aug 22;44(15):5167–79. doi: 10.1002/hbm.26456 (PMC10502647; doi:10.1002/hbm.26456)
Supplement: Supplementary file 1 — Data S1. Supporting Information. [file HBM-44-5167-s001.pdf]

The joint source maps of the parallel multilink joint ICA (jICA) are shown in the following figure.

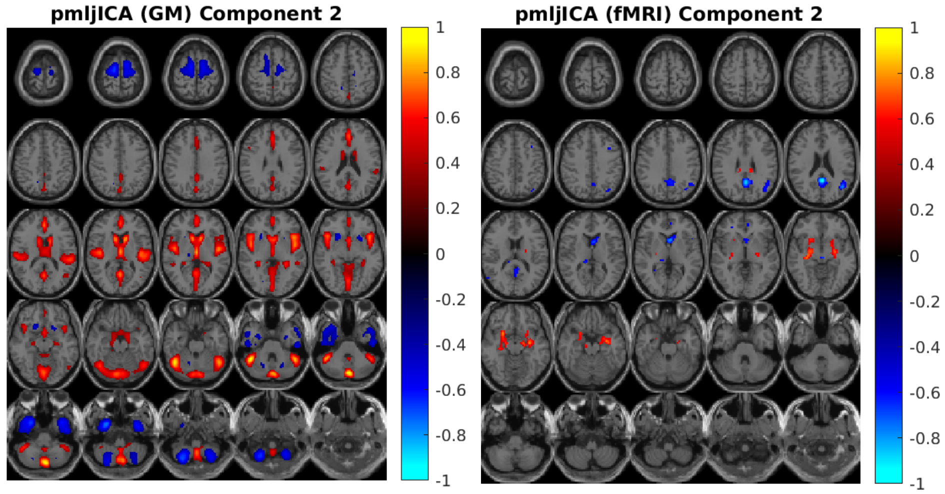

(a)

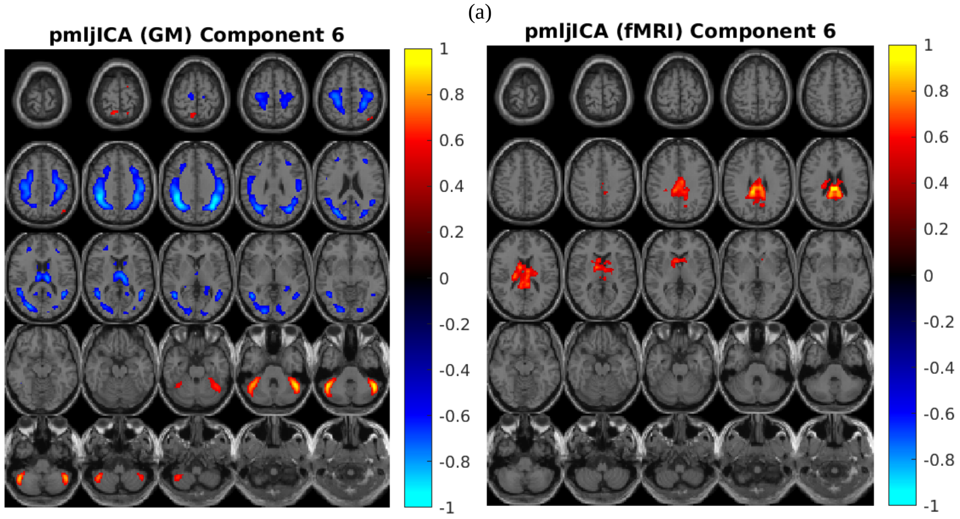

(b)

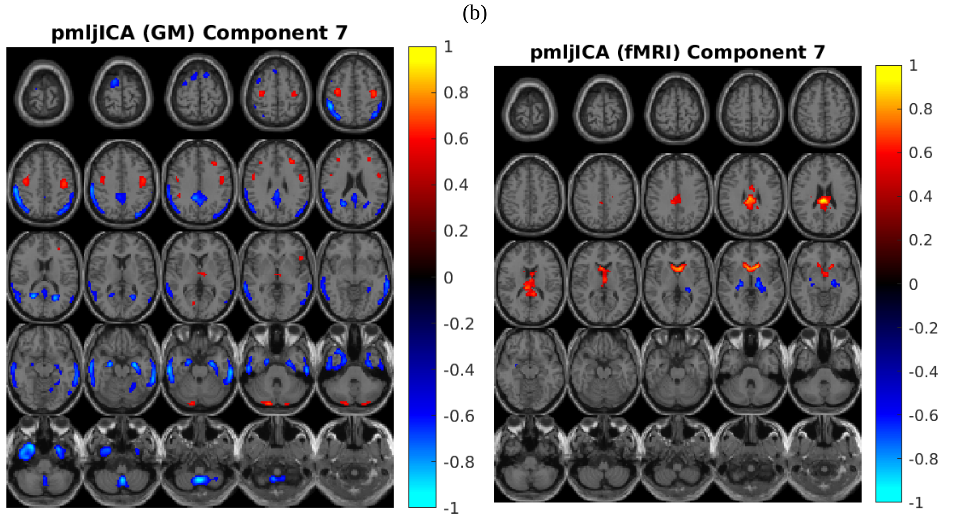

(c)

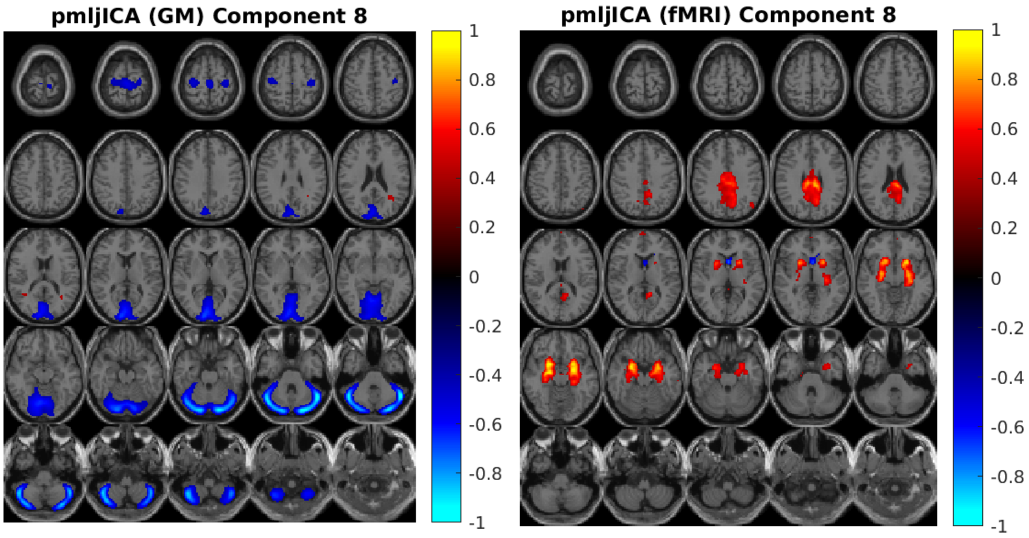
pmljICA (GM) Component 8
pmljICA (fMRI) Component 8
1
0.8
0.6
0.4
0.2
0
-0.2
-0.4
-0.6
-0.8
-1

(d)

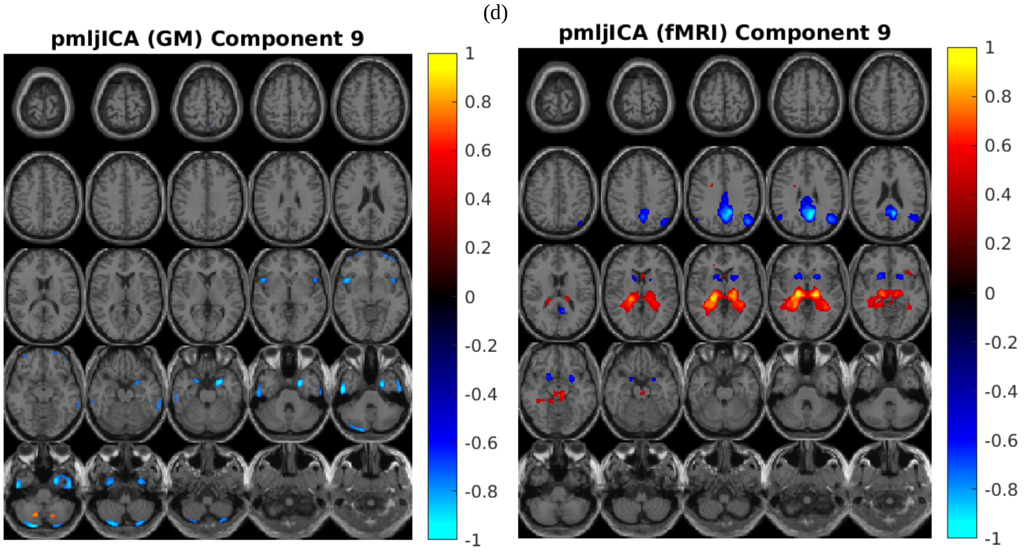
pmljICA (GM) Component 9
pmljICA (fMRI) Component 9
1
0.8
0.6
0.4
0.2
0
-0.2
-0.4
-0.6
-0.8
-1

(e)

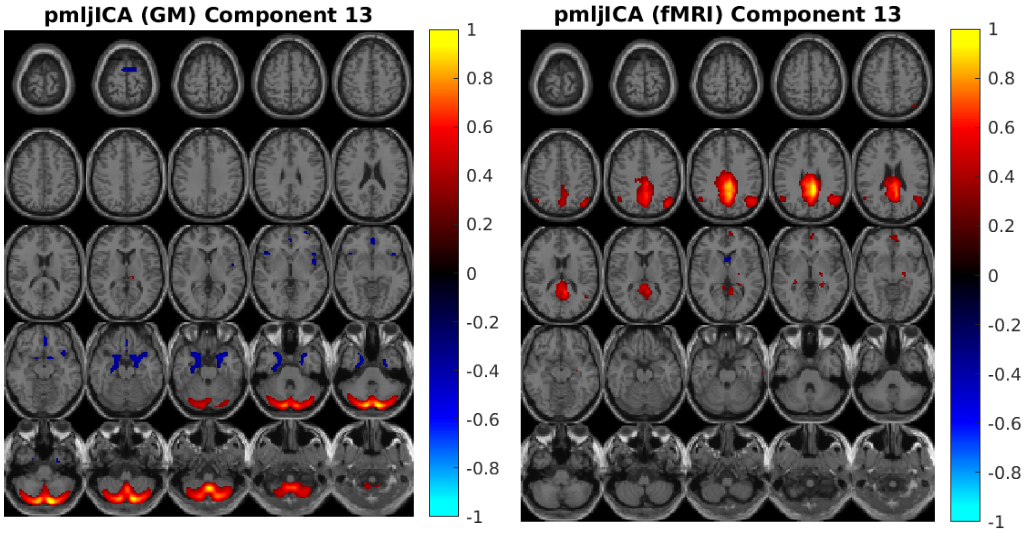
pmljICA (GM) Component 13
pmljICA (fMRI) Component 13
1
0.8
0.6
0.4
0.2
0
-0.2
-0.4
-0.6
-0.8
-1

(f)

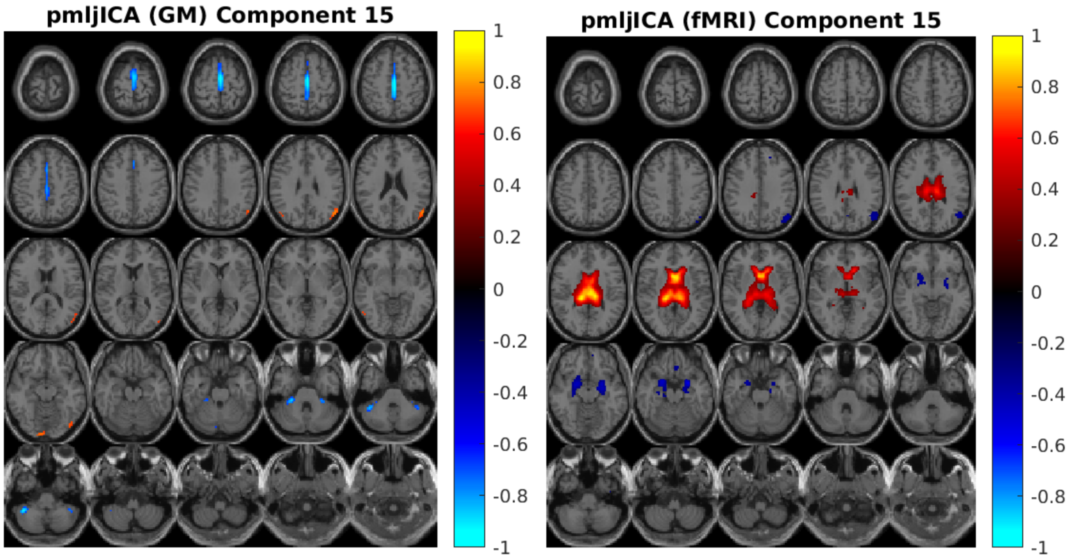

(g)

Figure-1: Joint source map for the Gray matter and rest fMRI. All of activated z score in the source maps are thresholded. The color bar indicates the color mapping of the activated voxels.

The following table summarized brain regions by domain, which are ordered by having volumes greater than 0.4 cm^3.

Table-I: Summarized activated brain regions by domain

**Joint ICs (pmljICA)**	**Brain Regions by Domain**		
Component 2	GM	Pos	**AU:** STG(8.3/4.9), TTG(1.2/1.0), MTG(1.1/0.3); **CC:** Insula(7.6/6.1), IFG(3.6/3.6), PHG(2.4/1.6), MeFG(2.2/2.6); **CB:** CL(5.7/6.3), DC(5.4/6.6), CT(2.6/2.8), ISLL(1.5/1.1), Tuber (1.4/1.9), Pyramis (0.9/0.8), Uvula (0.9/0.6); **SC:** Caudate (4.2/4.2), EN(3.5/3.6), TH(2.0/2.0); **DM:** AC(3.5/3.6), Precuneus (2.4/2.6), PC(1.3/2.0); **VI:** FG(3.1/3.8), LG(2.1/4.2); **SM:** PG(1.5/1.6)
		Neg	**AU:** ITG(3.1/3.4), MTG(2.8/3.3), STG(1.0/1.7); **CC:** MeFG(3.6/3.8), SFG(3.0/4.0), MFG (1.7/1.5), Uncus (0.8/2.3); **CB:** ISLL(2.4/4.0), CT(1.3/1.3); **SC:** EN(0.6/1.2), LN(0.5/0.3); **VI:** FG (0.6/1.0)
	fMRI	Pos	**CC:** PHG(2.0/1.2); **SC:** EN(1.7/2.2), LN(1.0/1.2); PC(0.1/0.0)
		Neg	**AU:** MTG(0.9/0.0), STG(0.5/0.1); **SC:** Caudate(0.9/0.0), TH(0.1/0.4); **DM:** Precuneus(2.4/0.9), PC(1.2/1.0), AC(0.4/0.0); **VI:** AG(0.6/0.0)
Component 6	GM	Pos	**CB:** CL(4.4/3.1), CT(3.3/3.9), Tuber (2.8/3.4), DC(0.9/0.6); **VI:** FG(0.4/0.1)
		Neg	**AU:** STG(3.3/3.1), MTG(2.6/4.0); **CC:** IPL(4.4/6.7), MFG(3.6/3.3), PHG(0.4/0.0), MeFG(0.3/0.6), IFG(0.2/0.4); **SC:** TH(3.0/2.2), EN(1.9/0.5), Caudate (0.7/0.3); **DM:** Precuneus (3.9/4.9), PC(0.6/0.2); VI: LG(1.7/1.8), AG(0.8/0.7); **SM:** PG(6.5/6.3), PL(0.4/0.3)
	fMRI	Pos	**SC:** EN(3.8/6.3), TH(1.5/1.7), Caudate(1.1/2.6), LN(0.1/0.4); **DM:** Precuneus(1.3/0.4), PC(0.9/1.1);

		Neg	
Component 7	GM	Pos	**CC:** IFG(1.2/0.3), MFG(1.0/0.6); **CB:** Uvula(0.4/0.6); **SC:** TH(0.6/0.2); SM: PG(0.9/0.1)
		Neg	**AU:** MTG(9.1/11.8), ITG(4.5/6.3), STG(1.6/6.6); **CC:** PHG(5.3/1.9), IPL(3.1/8.4), Uncus (2.6/2.8), SFG(1.1/2.6); **CB:** ISLL (1.5/1.2), CL(1.2/0.0), CT(1.0/1.0), DC(0.9/0.0); **SC:** EN (0.6/1.1); **DM:** Precuneus(2.8/1.5), PC(1.2/0.6); **VI:** FG(1.3/1.2), AG(1.3/0.8); **SM:** SPL(1.6/1.7)
	fMRI	Pos	**SC:** EN(3.4/3.3), Caudate(1.8/1.3), TH(0.5/0.8); **DM:** PC(0.4/0.4), AC(0.2/0.4)
		Neg	**SC:** EN(2.2/1.4), TH(1.5/0.1), LN(0.3/0.4)
Component 8	GM	Pos	
		Neg	**CC:** MeFG(1.1/1.4); **CB:** Tuber(7.1/6.7), CL(6.4/7.5), CT(5.4/5.8), ISLL(3.7/4.7), Pyramis (2.0/2.0), DC(10.9/11.0), Cuneus (10.2/15.4), Uvula (1.2/1.0); **DM:** PC(0.4/1.0), Precuneus(0.3/2.6); **VI:** LG(7.5/13.4), FG(2.2/4.2); **SM:** PG(0.6/0.3);
	fMRI	Pos	**CC:** PHG(7.5/5.4), Uncus (1.8/1.4), IFG(0.4/0.8); **SC:** EN(7.3/4.6), LN(5.5/4.7); **DM:** Precuneus(4.9/2.0), PC(3.1/1.0); **VI:** AG(0.5/0.0);
		Neg	**EN:** EN(0.4/0.2)
Component 9	GM	Pos	
		Neg	**AU:** ITG(1.2/1.3), MTG(1.2/1.3), STG(0.6/1.2); **CC:** Uncus(2.6/0.6), IFG(0.4/0.6), MFG(0.4/0.3), SFG(0.4/0.3); **CB:** ISLL(0.8/1.0), Pyramis (0.4/0.6), Tuber (0.2/0.7); **VI:** FG(0.2/0.4)
	fMRI	Pos	**SC:** EN(5.6/6.6), TH(5.4/5.5), LN(0.4/0.4)
		Neg	**AU:** MTG(1.2/0.1), STG(0.8/0.2); **CC:** IPL(1.3/0.0), PHG(0.4/0.4); **SC:** LN(1.9/1.7), EN(1.1/1.7), Caudate (0.3/0.7); **DM:** Precuneus (6.8/2.8), PC(1.9/0.8); **VI:** AG(2.6/0.3)
Component 13	GM	Pos	**CB:** ISLL(6.6/6.8), DC(3.9/5.4), Pyramis (3.2/3.1), Tuber (2.9/3.5), Uvula (2.4/2.3), CT(1.7/1.5), Pyramis of Vermis (0.4/0.2); **VI:** FG(0.1/0.4)
		Neg	**AU:** STG(1.8/1.1); **CC:** Uncus (2.2/3.3), IFG(1.9/1.2), PHG(1.7/4.4), SFG(0.7/0.1), MeFG(0.5/0.3), Insula (0.5/0.0);
	fMRI	Pos	**AU:** STG(1.7/0.3), MTG(1.3/0.1); **CC:** IPL(2.4/0.7), MeFG(1.3/0.2); **SC:** EN(4.0/3.0), TH(0.2/0.0); **DM:** PC(4.6/3.5), Precuneus (10.4/6.7); **VI:** AG(3.0/0.6)
		Neg	
Component 15	GM	Pos	**AU:** MTG(1.0/0.0); **VI:** AG(0.7/0.3)
		Neg	**CC:** MeFG(2.4/1.9), SFG (0.6/0.5); **CB:** CL(0.6/2.0), CT(0.3/1.2); **SM:** PL(2.5/1.2)
	fMRI	Pos	**CC:** PHG(0.5/0.3); **SC:** TH(8.3/8.8), Caudate (5.4/4.2), EN(14.8/16.1);
		Neg	**AU:** MTG(1.1/0.0); **CC:** PHG(2.6/2.5), Uncus (0.3/0.6); **SC:** EN(1.1/0.9), LN(0.4/0.7); **VI:** AG(1.6/0.0)

<u>Brain Domains</u>

AU: Auditory Domain
SC: Sub-cortical Domain
SM: Sensorimotor Domain
VI: Visual Domain
CC: Cognitive-control Domain
DM: Default-mode Domain
CB: Cerebellar Domain

<u>Brain Regions</u>

STG: Superior Temporal Gyrus
TTG: Transverse Temporal Gyrus
MTG: Middle Temporal Gyrus
ITG: Inferior Temporal Gyrus
IFG: Inferior Frontal Gyrus
PHG: Parahippocampal Gyrus
MeFG: Medial Frontal Gyrus
SFG: Superior Frontal Gyrus
MFG: Middle Frontal Gyrus
ISLL: Inferior Semi-Lunar Lobule
SPL: Superior Parietal Lobule
IPL: Inferior Parietal Lobule
CT: Cerebellar Tonsil

AG: Angular Gyrus
LG: Lingual Gyrus
PG: Postcentral Gyrus
PL: Paracentral Lobule
EN: Extra-Nuclear
LN: Lentiform Nucleus
FG: Fusiform Gyrus
PC: Posterior Cingulate
CL: Culmen
DC: Declive

Pos – Positive Activity
Neg – Negative Activity
